# Supplementary material for: Two distinct and separable processes underlie individual differences in algorithm adherence: Differences in predictions and differences in trust thresholds
Source: PLoS One. 2021 Feb 25;16(2):e0247084. doi: 10.1371/journal.pone.0247084 (PMC7906384; doi:10.1371/journal.pone.0247084)
Supplement: S1 Table — (DOCX) [file pone.0247084.s001.docx]

**S1 Table: Extended details of the questionnaire data.**

| **Questionnaire Item** | **Question** | **Answer type** | **n** | **test** | **corr.** | **p** |
| --- | --- | --- | --- | --- | --- | --- |
| **Demographics** | | | | | | |
| Age | *What is your age?* | Numerical Input | 103 | PR | -0.087 | 0.381 |
| Education | *What is your highest level of completed education?* | DDM | 103 | SR | 0.037 | 0.713 |
| Gender | *What is your gender?* | DDM (M/F/O) | 103 | OLS | N/A | 0.707 |
| Risk Aversion | *BRET, 10x10 grid hot version* | Incentivized Task | 103 | PR | -0.151 | 0.129 |
| **Smartphone and App usage** | | | | | | |
| Smartphone Usage | *How often do you use a smartphone?* | DDM (D/W/H/N) | 103 | SR^4^ | -0.019 | 0.575 |
| How often do you use the following types of apps on your smartphone: | | | | | | |
| Navigation Apps¹ | *Navigation Apps (ex: Google Maps)* | DDM (D/W/H/N) | 98 | SR^4^ | 0.14 | *0.085†* |
| Switch Default on Nav² | *How frequently do you select a different route than the route the app has at first selected for you?* | DDM (D/W/H/N) | 89 | SR^4^ | 0.006 | 0.477 |
| Transportation Apps¹ | *Transportation Apps (ex: Uber / Lyft)* | DDM (D/W/H/N) | 98 | SR^4^ | 0.03 | 0.384 |
| Banking Apps¹ | *Online Banking and Finance Apps* | DDM (D/W/H/N) | 98 | SR^4^ | 0.042 | 0.34 |
| Social Media Apps¹ | *Social Media Apps (ex: Facebook / Instagram)* | DDM (D/W/H/N) | 98 | SR^4^ | 0.014 | 0.445 |
| Dating Apps¹ | *Dating Apps (ex: Tindr, Bumble, Grindr)* | DDM (D/W/H/N) | 98 | SR^4^ | 0.03 | 0.383 |
| Housing Apps¹ | *Short-term Housing Apps (ex: AirBnB)* | DDM (D/W/H/N) | 98 | SR^4^ | 0.116 | 0.127 |
| Fitness Apps¹ | *Personal Fitness Apps* | DDM (D/W/H/N) | 98 | SR^4^ | -0.012 | 0.546 |
| **Online video suggestions** | | | | | | |
| Suggestions Followed | *I typically follow [online video] suggestions:* | SDS (Never - Always) | 103 | PR^4^ | 0.094 | 0.173 |
| Suggestions Useful^3^ | *I typically find [online video] suggestions to be:* | SDS (Useful - Useless) | 103 | PR^4^ | -0.004 | 0.518 |
| **Attitudes Towards Computers** | | | | | | |
| Importance Smartphone | *How important is your smartphone in your daily life?* | SDS (Unimportant - Unmissable) | 103 | PR^4^ | 0.026 | 0.397 |
| Trustworthiness Computers | *In my daily life, I typically find computers to be:* | SDS (Unreliable - Trustworthy) | 103 | PR^4^ | 0.247 | 0.006** |
| Likelihood Mistakes | *In most tasks, computers are more likely to make* | SDS (Agree - Disagree) | 103 | PR^4^ | 0.163 | *0.05†* |
|  | *mistakes than humans* |  |  |  |  |  |
| Trust after mistake | *After seeing a computer make a mistake,* | SDS (Agree - Disagree) | 103 | PR^4^ | 0.012 | 0.451 |
|  | *I will never trust it again* |  |  |  |  |  |
| **Would you trust a computer to...** | | | | | | |
| Finances | *...manage your personal finances?* | SDS (Not at all - Very much) | 103 | PR^4^ | 0.171 | 0.042* |
| Invest | *...invest into the stock market on your behalf?* | SDS (Not at all - Very much) | 103 | PR^4^ | 0.219 | 0.013* |
| Fitness | *...manage your personal fitness plan?* | SDS (Not at all - Very much) | 103 | PR^4^ | 0.135 | *0.087†* |
| Meals | *...plan your meals?* | SDS (Not at all - Very much) | 103 | PR^4^ | 0.127 | *0.1†* |
| Diagnose | *...diagnose health problems?* | SDS (Not at all - Very much) | 103 | PR^4^ | 0.185 | 0.031* |
| Driving | *...drive your vehicle for you (such as a self-driving car)?* | SDS (Not at all - Very much) | 103 | PR^4^ | 0.208 | 0.018* |

*Notes: ^1^Question only shown if smartphone usage not equal to never. ^2^Question only shown if navigation app usage not equal to never. ^3^Recoded variable (inverted). ^4^One-sided test. Abbr: DDM = Drop Down Menu, D/W/H/N = Daily / Weekly / Hourly / Never, M/F/O = Male / Female / Other, SDS = Semantic Difference Scale, PR = Pearson's R, SR = Spearman's Rho. Significance: ^†^p<0.1, ^*^p<0.05, ^**^p<0.01, ^***^p<0.001*
